# Penalization and shrinkage methods produced unreliable clinical prediction models especially when sample size was small

**DOI:** 10.1016/j.jclinepi.2020.12.005

**Published:** 2021-04

**Authors:** Richard D. Riley, Kym I.E. Snell, Glen P. Martin, Rebecca Whittle, Lucinda Archer, Matthew Sperrin, Gary S. Collins

**Affiliations:** a Centre for Prognosis Research, School of Medicine, Keele University, Staffordshire, UK, ST5 5BG; b Division of Informatics, Imaging and Data Science, Faculty of Biology, Medicine and Health, University of Manchester, Manchester Academic Health Science Centre, Manchester, UK; c Nuffield Department of Orthopaedics, Centre for Statistics in Medicine, Rheumatology and Musculoskeletal Sciences, University of Oxford, Oxford, UK, OX3 7LD; d NIHR Oxford Biomedical Research Centre, John Radcliffe Hospital, Oxford, OX3 9DU, UK

**Keywords:** Risk prediction models, Penalization, Shrinkage, Overfitting, Sample size

## Abstract

**Objectives:**

When developing a clinical prediction model, penalization techniques are recommended to address overfitting, as they shrink predictor effect estimates toward the null and reduce mean-square prediction error in new individuals. However, shrinkage and penalty terms (‘tuning parameters’) are estimated with uncertainty from the development data set. We examined the magnitude of this uncertainty and the subsequent impact on prediction model performance.

**Study Design and Setting:**

This study comprises applied examples and a simulation study of the following methods: uniform shrinkage (estimated via a closed-form solution or bootstrapping), ridge regression, the lasso, and elastic net.

**Results:**

In a particular model development data set, penalization methods can be unreliable because tuning parameters are estimated with large uncertainty. This is of most concern when development data sets have a small effective sample size and the model's Cox-Snell $R^{2}$ is low. The problem can lead to considerable miscalibration of model predictions in new individuals.

**Conclusion:**

Penalization methods are not a ‘carte blanche’; they do not guarantee a reliable prediction model is developed. They are more unreliable when needed most (i.e., when overfitting may be large). We recommend they are best applied with large effective sample sizes, as identified from recent sample size calculations that aim to minimize the potential for model overfitting and precisely estimate key parameters.

## Highlights

• When developing a clinical prediction model, penalization and shrinkage techniques are recommended to address overfitting.
• Some methodology articles suggest penalization methods are a ‘carte blanche’ and resolve any issues to do with overfitting.
• We show that penalization methods can be unreliable, as their unknown shrinkage and tuning parameter estimates are often estimated with large uncertainty.
• Although penalization methods will, on average, improve on standard estimation methods, in a particular data set, they are often unreliable.
• The most problematic data sets are those with small effective sample sizes and where the developed model has a Cox-Snell $R^{2}$ far from 1, which is common for prediction models of binary and time-to-event outcomes.
• Penalization methods are best used in situations when a sufficiently large development data set is available, as identified from sample size calculations to minimize the potential for model overfitting and precisely estimate key parameters.
• When the sample size is adequately large, any of the studied penalization or shrinkage methods can be used, as they should perform similarly and better than unpenalized regression unless sample size is extremely large and $R_{app}^{2}$ is large.

**What is new?**

**Key Findings**
• When developing a clinical prediction model, penalization techniques are recommended to address overfitting; however, they are not a ‘carte blanche’.

**What this adds to what was known?**
• Although penalization methods will, on average, improve on standard estimation methods, in a particular data set, they can be unreliable, as their unknown shrinkage and tuning parameter estimates are often estimated with large uncertainty.
• The most problematic data sets are those with small effective sample sizes and where the developed model has a Cox-Snell *R^2^* far from 1, which is common for prediction models of binary and time-to-event outcomes.

**What is the implication and what should change now?**
• Penalization methods are best used in situations when a sufficiently large development data set is available, as identified from sample size calculations to minimize the potential for model overfitting and precisely estimate key parameters.
• When the sample size is adequately large, any of the studied penalization or shrinkage methods can be used, as they should perform similarly and better than unpenalized regression unless sample size is extremely large and *R^2^_app_* is large.

## 1 Introduction

In health care, diagnosis and prognosis may be informed by statistical models that predict disease presence and outcome occurrence in individuals [1]. Such models are broadly known as clinical prediction models [2] and are often developed using a multivariable regression framework (e.g., logistic, survival, or linear regression), which provides an equation to estimate an individual's outcome probability (for binary or time-to-event outcomes) or outcome value (for continuous outcomes) conditional on values of multiple variables (predictors).

When estimating regression models using a particular data set, conventional estimation techniques are often used, in particular ordinary least squares or standard maximum likelihood estimation. However, these tend to give model equations that are overfitted to the development data set and so produce too extreme predictions when applied in new individuals, that is, some predicted outcome values lie too far from the overall mean. For example, using standard maximum likelihood estimation when developing a logistic regression model can give predicted probabilities too close to 0 for low-risk individuals and too close to 1 for high-risk individuals. The problem of overfitting will usually increase as the sample size of the development data decreases, the number of candidate predictors increases, and (for binary or time-to-event outcomes) the number of outcome events decreases.

Penalization and shrinkage methods have been proposed to resolve overfitting concerns. These include uniform shrinkage estimated via bootstrapping, ridge regression, the least absolute shrinkage and selection operator (lasso), and elastic net [[3], [4], [5], [6]]. Penalization techniques shrink (in fact, introduce bias to) the estimated predictor effect estimates (i.e., odds ratios, hazard ratios, or mean differences) toward the null. Compared with standard estimation methods, this reduces the variance of the developed model's predictions in new individuals, thereby reducing the mean-square error of the predictions. For example, in a logistic regression model, penalization methods will shrink predictor effects (odds ratios) toward 1, so that predicted probabilities in new individuals show less variability (i.e., are pulled away from 0 and 1, toward the mean outcome probability in the data set).

Penalization techniques are thus recommended as essential tools for prediction model development [3,[7], [8], [9], [10], [11], [12]], especially for situations where the effective sample size is low (and thus potential magnitude of overfitting using standard methods is high) [13]. For example, Pavlou et al. conclude that “penalized regression is a flexible shrinkage approach that is effective when the EPV is low (<10)” [8], and Ambler et al. note that “the performance of ridge and lasso in our simulations suggests that it is possible, with care, to develop a risk model when the EPV is as low as 2.5” [7]. Given such recommendations, we are concerned that applied researchers might view penalization methods as a ‘carte blanche’ to develop a prediction model regardless of the size of the data set available for model development. Indeed, previous simulations suggest that although penalization may indeed be effective on average, it may fail in the particular data set being used for model development [12,14].

In this article, we build on previous statistical articles [12,14], to highlight this issue to a broad audience. We use applied examples and graphical displays to show that shrinkage and tuning parameters in penalized regression are typically estimated with large uncertainty. We demonstrate how this problem increases as the effective sample size reduces (i.e., when development data sets have smaller numbers of participants or events, relative to the number of candidate predictors) [[15], [16], [17]] and that the consequence is miscalibration and poor performance when the model is applied to new individuals. The impact on blood pressure predictions is demonstrated at the individual level, and our empirical examples are reinforced by analytic reasoning and a small simulation study. Section 2 describes our methods, Section 3 reports our results and applied examples, and Section 4 concludes with discussion and recommendations.

## 2 Methods

Penalization methods for prediction model development are now described, followed by details of our applied examples and simulation study for evaluating their performance.

### 2.1 Shrinkage and penalization methods

Perhaps the simplest penalization method is where a uniform (linear and global) shrinkage factor (S) is used to shrink the predictor effects estimated from standard (unpenalized) maximum likelihood estimation. For example, a modified logistic regression model with shrunken predictor effects can be obtained by

$$\text{ln}\left(\frac{{\hat{p}}_{i}}{1-{\hat{p}}_{i}}\right)=\alpha^{*}+S\left({\hat{\beta }}_{1}X_{1i}+{\hat{\beta }}_{2}X_{2i}+{\hat{\beta }}_{3}X_{3i}+\ldots \right)$$

where S is a shrinkage value between 0 and 1 and used to uniformly adjust the predictor effects (${\hat{\beta }}_{1},{\hat{\beta }}_{2},\ldots )$ estimated from standard maximum likelihood and $\alpha^{*}$ is the updated intercept, estimated after determining and applying S to ensure that the calibration-in-the-large is correct (i.e., that the sum of predicted probabilities equals the overall proportion of observed events).

The true S is that which minimizes the expected mean-square error of predictions from the model when applied to the same population as that which the development data set is sampled from. However, S is an unknown parameter and so must be estimated, for example, using the heuristic solution of Van Houwelingen and Le Cessie (given in Appendix) [18], or via bootstrapping as described elsewhere [1,2,19,20]. Riley et al. showed the estimate of S depends on $R_{app}^{2}$, which is the apparent (‘app’) value of the Cox-Snell $R^{2}$ (a measure of proportion of variance exampled by the model in the development data set) [21].

Rather than using a postestimation uniform shrinkage to penalize the regression coefficients from standard maximum likelihood estimates, other approaches are available that penalize during the model estimation itself [12]. In this article, we focus on three popular penalized regression methods: ridge regression [4,6], the lasso [3], and elastic net [5]. Such penalization approaches include a term λpen(β), where pen(β) is a particular penalty term and λ is a nonnegative tuning parameter, which controls the amount of shrinkage. Further details of the penalty terms for each approach are given in Appendix. A value of λ = 0 corresponds to no shrinkage (i.e., applying the standard maximum likelihood estimator when fitting a model such as logistic regression). The penalty factor for elastic net or the lasso is defined such that it can shrink predictor effects to zero and hence allows the exclusion of some predictors. The penalty term in ridge regression sets many of the predictor coefficients close to zero, but never exactly to zero.

As explained for the uniform shrinkage factor S, the true value of the tuning parameter λ is the one that minimizes the mean-square error of model predictions in the target population. However, λ is unknown and so is often estimated from the development data set using either *K*-fold cross-validation, repeated *K*-fold cross-validation, or bootstrap *K*-fold cross-validation [22]. Larger uncertainty in the value of λ leads to more uncertainty in the model's actual predictive accuracy [23].

### 2.2 Examples to illustrate uncertainty of uniform shrinkage estimate

Three prediction models are used to illustrate uniform shrinkage and the potential for uncertainty in the estimate of S. First, we developed two models for predicting systolic blood pressure (SBP) at 1 year in patients diagnosed with hypertension. The development data set was based on a subset of Riley et al. [24], and to contrast models with different $R_{app}^{2}$ values, we developed models separately in those considered low risk (model A, 262 participants with no comorbidities) or high risk (model B, 253 participants with comorbidities) for developing cardiovascular disease. Seven predictors measured at baseline were used in the modeling: SBP (mmHg), diastolic blood pressure (mmHg), body mass index (kg/m^2^), age (years), sex (female = 0, male = 1), current smoker (yes = 1, no = 0), and antihypertensive treatment (yes = 1, no = 0).

Second, we used data from 654 children to develop a model (model C) to predict log-transformed forced expiratory volume (in liters) using four predictors: age (years), height (inches), sex (female = 0, male = 1), and current smoker (yes = 1, no = 0). The data were obtained from http://biostat.mc.vanderbilt.edu/DataSets and originally come from Rosner [25].

### 2.3 Simulation study to examine uncertainty of tuning parameter estimates

Our simulation study to examine the degree of instability of the various penalization methods is now described. The corresponding R code is provided at https://github.com/gscollins1973.

#### 2.3.1 Scenarios

We considered 10 simulation scenarios. All scenarios had an outcome event proportion of 0.5 (50%) but varied in the sample size from *N* = 100 to *N* = 1,000 (in steps of 100), corresponding to an events-per-parameter value of 2.5 (for *N* = 100; 50 outcome events) to 25 (for *N* = 1,000; 500 outcome events). The scenarios are a pragmatic choice, to cover a range of events per parameter and to differ from those elsewhere [12,14], but we recognize that they do not reflect all possible model development settings.

#### 2.3.2 Data generation

For each scenario, 500 data sets of the chosen sample size were generated. Each data set was simulated containing a binary outcome and values of 20 continuous predictors for each participant. First, values of the 20 continuous predictors were simulated using a multivariate normal distribution with mean 0 and variance 1: five weakly correlated true predictors (pairwise correlation = 0.1) and 15 uncorrelated noise predictors (pairwise correlation = 0). Then, the true outcome (Y) was generated conditional on an underlying logistic regression model based on all 20 predictors (equation given in appendix).

#### 2.3.3 Model development

To each of the 500 simulated data set in each scenario, six different methods were used to develop a logistic regression model based on the 20 predictors: three penalized regression methods (ridge regression, elastic net, and lasso), two uniform shrinkage methods (heuristic shrinkage or bootstrap shrinkage), and, for comparison, standard (unpenalized) regression. The tuning parameter (λ) for the ridge regression, elastic net, and lasso was estimated using 5-fold cross-validation, using the cv.glmnet function from the glmnet package in R [26], to minimize the deviance. In addition to 5-fold cross-validation, we also investigated whether using bootstrapping would improve stability for ridge regression. In each bootstrap sample (total of 200 bootstrap samples), 5-fold cross-validation was used to select the tuning parameter, and the median λ over the 200 bootstrap samples was taken forward to develop the model.

#### 2.3.4 Model validation

To evaluate the performance of each developed prediction model, a validation data set (*N* = 5,000; 2,500 outcome events) was created using the same data generating procedure, giving a much larger effective sample size than the recommended 100 to 250 outcome events for validating a prediction model [27,28]. Each developed model was evaluated in this independent validation data set by calculating the c-index, Nagelkerke R^2^, calibration-in-the large, and the calibration slope.

#### 2.3.5 Summarizing simulation results

For each type of model (e.g., uniform shrinkage, lasso, elastic net, ridge regression), in each scenario, the key findings are the average model performance and, in particular, the variability in model performance across the 500 developed models in each scenario and also the variability in tuning parameter estimates. To illustrate this, boxplots are presented for each scenario.

## 3 Results

The key findings from our applied examples and simulation study are now described.

### 3.1 Uncertainty in uniform shrinkage estimate: findings from applied examples

Consider the three models to predict a continuous outcome introduced in Section 2.2. The corresponding unpenalized model equations are shown in Table 1 and cover situations with small (model A), medium (model B), and large (model C) values for $R_{app}^{2}$ of 0.23, 0.56, and 0.81, respectively. For each model, Table 1 shows the mean bootstrap estimate of S together with a 95% confidence interval for S derived from the 2.5th and 97.5th percentile values of the bootstrap samples. Our ‘best guess’ of the true S is the mean estimate across 1,000 bootstrap samples, but the width of the distribution of the 1,000 values reveals the uncertainty in this choice. If the width is narrow, it gives more assurance that the mean estimate is suitable; however, if the width is wide, the mean estimate may be far from the true value of S for the target population.

**Table 1:** Three prediction models developed using linear regression, with summary of model performance and bootstrap uniform shrinkage estimate

Model	Outcome	Model equation derived using ordinary least squares estimation (i.e., before any shrinkage)	Number of patients/predictor parameters	$R_{app}^{2}$	Uniform shrinkage (S) estimate from 1,000 bootstrap samples (95% confidence interval)
A	Systolic blood pressure (SBP) (low CVD risk population)	28.10 + 0.46∗SBP + 0.41∗DBP + 0.013∗BMI + 0.45∗age − 2.05∗sex − 17.81∗treat − 2.08∗smoker	262/7 = 37	0.23	0.94 (0.77 to 1.18)
B	Systolic blood pressure (SBP) (high CVD risk population)	−12.69 + 0.94∗SBP + 0.21∗DBP −0.001∗BMI + 0.06∗age + 1.72∗sex − 1.04∗treat + 0.17∗smoker	253/7 = 36	0.56	0.98 (0.87 to 1.10)
C	ln(FEV)	−2.07 + 0.02∗age + 0.04∗height + 0.03∗sex + 0.05∗smoker	654/4 = 164	0.81	1.00 (0.96 to 1.04)

DBP, diastolic blood pressure; BMI, body mass index; CVD, cardiovascular disease.

**Fig. 1:**
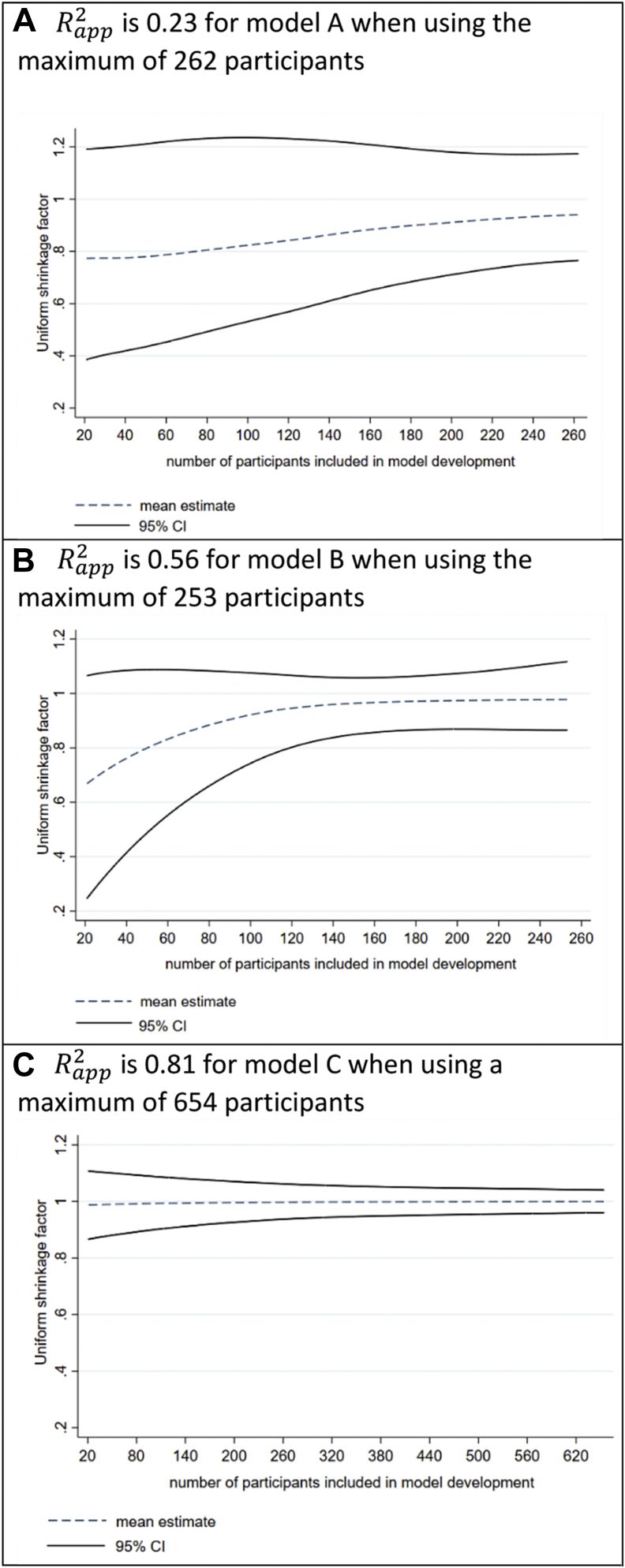
The mean estimate and 95% confidence interval of the uniform shrinkage factor (S) as derived from 1,000 bootstrap samples, across different sample sizes for developing models A, B, and C as described in Table 1. Curves are created using a lowess smoother.

For model A, which has the smallest $R_{app}^{2}$ value (0.23) in the full development data set and contains seven predictor parameters, the bootstrap mean estimate of S is 0.94, which reflects low overfitting because of the larger number of 37 individuals per predictor parameter. Despite this, the range of observed bootstrap values for S is reasonably wide, with 95% of values lying between 0.77 and 1.18. Thus, we are not very confident about what the actual (true) shrinkage factor should be. Hence, even in an example where overfitting is expected to be low, the true shrinkage factor is very difficult to ascertain from the data set at hand. Fig. 1A shows that the uncertainty of S increases as the number of participants in the development data set decreases and as the corresponding mean estimate of S moves closer to zero. For example, when refitting model A using a random subset of 100 participants, the mean bootstrap estimate of S is 0.82 and 95% of values fall between 0.52 and 1.27. When using a subset of only 50 participants, the mean estimate of S is again smaller (0.78) and the 95% range of values wider (0.43 to 1.21). Thus, as the required level of shrinkage increases, the uncertainty in the shrinkage factor also increases.

Now consider model B, which was developed using 253 individuals and seven predictors. These are similar numbers to model A, but model B has a much larger $R_{app}^{2}$ (0.56) in the full development data set, a mean shrinkage estimate (0.98) closer to 1, and a narrower range of values for S (95% interval: 0.87 to 1.10). To reduce sample size, we sequentially removed one individual randomly at a time and repeated the modeling process; this shows again that the mean bootstrap estimate of S reduces and the observed range of values widens (Fig. 1B), especially as the sample size reduces below 100 individuals. For example, with only 50 individuals, the mean estimate of S drops to 0.84 and 95% of bootstrap values lie between 0.52 and 1.10.

Model C has an $R_{app}^{2}$ of 0.81 in the full development data set, much larger than those for models A and B. The mean shrinkage estimate is 1, and the 95% interval is very narrow (Fig. 1C); essentially, there is strong evidence that shrinkage of predictor effects is not required. This is not surprising as there are only four predictor parameters and 654 participants. Indeed, even if we developed the model in a reduced set of 20 randomly selected participants, the mean shrinkage estimate is still close to 1 and the 95% interval remains quite narrow (about 0.9 to 1.1).

Notice that, in these examples, there is less uncertainty in the value of S when the estimate is closer to 1, that is, settings where overfitting is less of a concern. Furthermore, the estimate of S is closer to 1 in settings where $R_{app}^{2}$ is closer to 1 (this is also shown analytically in the Appendix). Most prediction models do not have $R_{app}^{2}$ values close to 1. In particular, for binary and time-to-event outcomes, the value of $R_{app}^{2}$ will often be much lower than 1, as the maximum value is bounded below 1 [16]. Consequently, uniform shrinkage estimates will often be far from 1 (toward 0) in models such as logistic and Cox regression, especially when the sample size is small and the number of predictor parameters is large.

### 3.2 Importance of estimating shrinkage precisely: illustration using model A

**Fig. 2:**
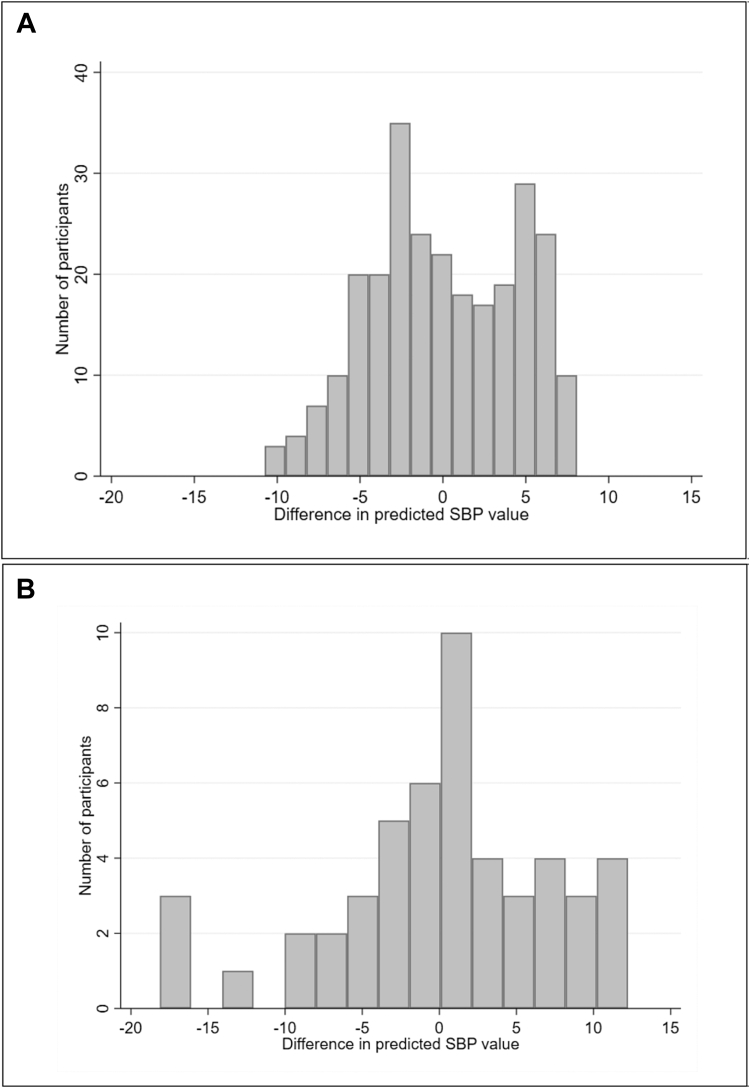
Difference in predicted systolic blood pressure (SBP) values in mmHg, when using the lower or upper bound of the bootstrap-derived 95% confidence interval for the shrinkage factor (S) to revise model A after (A) using 262 participants and (B) using 50 participants for model development.

To illustrate the importance of estimating S precisely, we applied uniform shrinkage to revise the model A equation shown in Table 1. Based on the full development data set of 262 participants, bootstrap shrinkage suggests an S of 0.94 with a 95% confidence interval between 0.77 and 1.18 (Table 1). We compared predictions after applying shrinkage values of 0.77 and 1.18. The difference in predictions was generally within 7–8 mmHg for most participants, and only seven (2.7%) of the participants had a difference > 10 mmHg (Fig. 2A).

Next, we reduced the development data set to a random sample of 50 participants and re-estimated the equation for model A. Because of the smaller sample size, the bootstrap approach gave a lower estimate of 0.78 for S, with a wider 95% confidence interval of 0.43 to 1.21. We compared predictions after applying shrinkage values of 0.43 and 1.21. Compared with when using all 262 participants, the distribution of differences in predictions was much wider, and now 10 (25%) of the participants had absolute differences > 10 mmHg; three participants even had a >15 mmHg absolute difference (Fig. 2B). Hence, the larger the uncertainty in the shrinkage factor, the larger the uncertainty in predicted values for individuals, and so the greater the concern that model predictions may not be reliable for practice.

### 3.3 Uncertainty in uniform shrinkage and penalized regression methods: findings from simulation study

We now discuss the uncertainty when fitting penalized regression models, as identified by our simulation study of uniform shrinkage, ridge regression, the lasso, and elastic net.

#### 3.3.1 Comparison of uncertainty in bootstrap and heuristic shrinkage estimates of S

For the uniform shrinkage approach, the simulations confirmed the findings identified from the applied examples. In particular, there was more variability in the estimate of uniform shrinkage with more overfitting (i.e., when S was further from 1 toward 0), as shown in Supplementary material Figure S1. In addition, the simulation results showed considerably less variability in the bootstrap estimate of S than in the heuristic shrinkage estimate of S when using small sample sizes for model development and when the required shrinkage is large (S<0.8) (Supplementary material Figure S1). When the sample sizes were larger such that required shrinkage is smaller (S>0.8), the bootstrap and heuristic approaches were much more similar in their distribution of S estimates. This again suggests that estimates of S are more stable when the overfitting is small (i.e., S is closer to 1).

#### 3.3.2 Uncertainty in tuning parameter estimates and prediction model performance

**Fig. 3:**
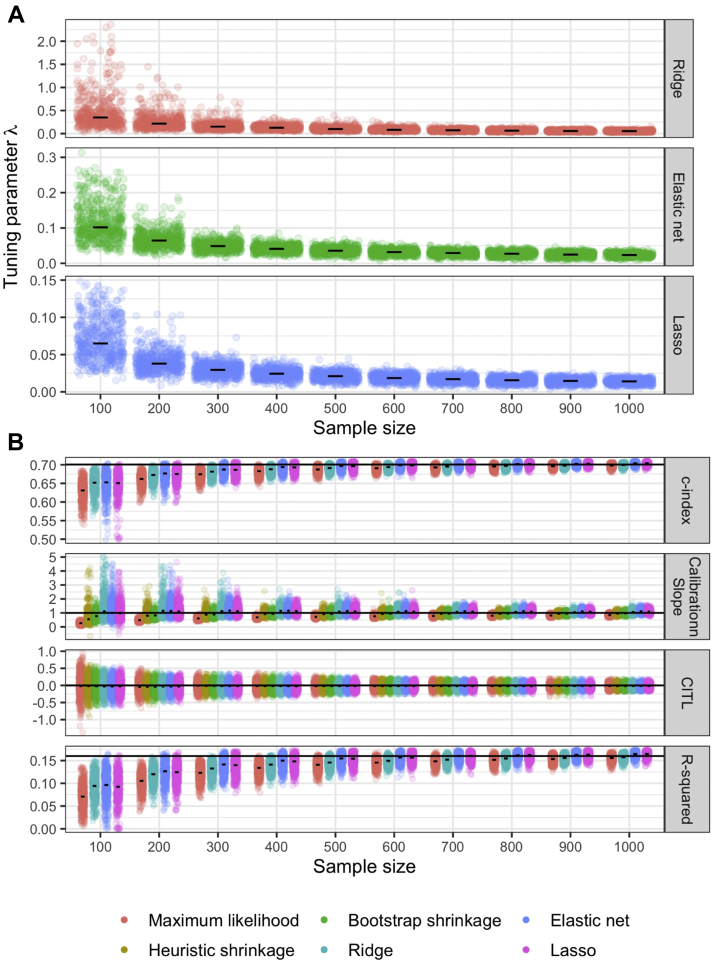
Median values (short horizontal lines) and scatter plots showing variability in (A) tuning parameter estimate (λ) and (B) predictive performance of the developed model in the large validation data, for various methods across varying sample sizes for model development; for each sample size, 500 data sets were simulated as described in Section 2.3, and for each data set, a model was developed for each method with (A) tuning parameter estimated, and then (B) predictive performance tested. In (B), the long horizontal lines are the large sample performance values. CITL = calibration-in-the-large; Horizontal spread within each sample size grouping is just random jitter to aid display; c-index is not shown for heuristic or bootstrap shrinkage, as these methods do not change the ranking of predictions, and thus the c-index is the same as maximum likelihood estimation.

Figure 3A illustrates the variability in the estimation of the tuning parameter, λ, in our simulation scenarios for each of the three penalized regression approaches. For all three approaches, the smaller the sample size, the more uncertainty in the estimate of λ. Variability in the estimate of the λ was reduced using bootstrap 5-fold cross-validation, compared with standard 5-fold cross-validation for ridge regression (Supplementary Figure S5). There was negligible difference between 5-fold and 10-fold cross-validation (supplementary Tables S1 to S3).

Importantly, Figure 3B shows that more variability in λ led to more variability in the predictive performance of the developed models when tested in the large validation data set (also see Supplementary Material Figures S2, S3 and S5). Even with the best model development approaches, there was considerable variability in a model's predictive performance on validation, especially when the development data set had a small sample size. In particular, for every model, there was large variability in the calibration slope on validation, and the slope was often very different to 1 (i.e., indicative of miscalibration), with some methods even giving a range of about 0 to 5 in the smallest sample sizes (Fig. 3B). Therefore, despite their intention, the various penalization and shrinkage methods did not provide a ‘carte blanche’ that ensured a reliable model when applied in new individuals.

Large development sample sizes were needed to reduce the variability in both the estimate of λ and predictive performance on validation, for the penalization and shrinkage methods to give a more reliable model for practice (i.e., as the model development data set became larger, the methods produced models with calibration slopes more consistently close to 1 when applied in new data). In that situation, the various penalization and shrinkage methods performed similarly (Fig. 3B, Supplementary material Figure S5), with an average calibration slope estimate close to 1 and reasonably narrow variability (e.g., 0.8 to 1.2). Other measures were also more stable, such as $R^{2}$, the c-index and the calibration-in-the-large (Fig. 3B), and we would expect this to also hold for other measures such as the integrated or estimated calibration index [29,30].

## 4 Discussion

Our findings emphasize the potential for large uncertainty of shrinkage and tuning parameter estimates used within penalization methods when developing prediction models. Although penalization methods are recommended because they will improve on standard estimation methods *on average*, in a particular data set, they can be unreliable. The most problematic data sets are those with small effective sample sizes and where the developed model has an $R_{app}^{2}$ far from 1, which is common for prediction models of binary and time-to-event outcomes [16]. Such uncertainty might lead to considerable miscalibration of predictions when the model is applied to individuals outside the development data set.

A limitation of our work is that the simulation study scenarios were pragmatic, and so do not cover every possible type of model development data set and setting. However, the findings echo related simulation studies by Van Houwelingen [12] and Van Calster et al. [14]. Furthermore, our simulation scenarios differ from those used in these articles, and we also examined the reduction in uncertainty in estimating tuning parameters over k-fold cross-validation using repeated k-fold cross-validation and bootstrap k-cross-validation and assessed how this impacts on reducing uncertainty in model performance measures. We also demonstrated the issues using analytic reasoning and applied examples, to showcase the problem to a wider audience.

### 4.1 Recommendations

In model development data sets with large potential for overfitting, the uncertainty about the true magnitude of penalization will often be very large, and thus there is actually no guarantee they will improve calibration of predictions in new individuals. Indeed, in situations where penalization methods are most needed, they are more likely to be unreliable. Hence, we recommend that researchers are best applying penalization methods to develop a prediction model when the effective sample size is large, such that the amount of shrinkage and penalization is anticipated to be small. In recent guidance [[15], [16], [17]], we show how researchers can base sample size requirements on a targeted uniform shrinkage factor of at least 0.9, such that the magnitude of global shrinkage is desired to be 10% or less. In such situations, penalization methods will have narrower uncertainty about the estimated tuning and shrinkage parameters, such that developed models are more likely to be reliable (well calibrated) when applied to new individuals. This idea is supported by Figure 1, which shows that the range of observed bootstrap values for the shrinkage factor (S) becomes more acceptably narrow for sample sizes where the mean estimate of S is between 0.9 and 1.0. Conversely, where the estimate of S is less than 0.9, the variability in bootstrap values may escalate quite rapidly. Even when S is estimated close to 0.9 or above, the uncertainty may be surprisingly large still in some applications (e.g., see confidence interval for S for model A, Table 1). Sample size should also be large enough to estimate overall model fit precisely and key parameters such as the model intercept [[15], [16], [17]].

Another key finding is that in smaller samples, bootstrapping resulted in less variability in estimates of shrinkage than other methods, including the heuristic estimate of uniform shrinkage and k-fold cross-validation estimate of tuning parameters in penalized regression. However, in situations where sample size is larger, the various methods were similar in terms of variability of shrinkage (and subsequent predictive performance on validation). Hence, when the sample size is adequate, we recommend that any of the studied penalization or shrinkage methods can be used, as they should perform similarly and better than unpenalized regression unless sample size is extremely large and $R_{app}^{2}$ is large (e.g., as in model C).

## 4.2 Summary

In summary, penalization and shrinkage methods should not be viewed as a solution to small effective samples sizes for prediction model development. We recommend they are best applied to develop models in situations where a sufficiently large development data set is available, to minimize the potential for model overfitting, improve the precision of model parameter estimates (including shrinkage and tuning parameters), and thus give more robust prediction models for clinical practice.
