# Supplementary material for: Penalization and shrinkage methods produced unreliable clinical prediction models especially when sample size was small
Source: J Clin Epidemiol. 2021 Apr;132:88–96. doi: 10.1016/j.jclinepi.2020.12.005 (PMC8026952; doi:10.1016/j.jclinepi.2020.12.005)
Supplement: Supplementary Material [file mmc1.docx]

**APPENDIX:**

**Further description on shrinkage and penalisation methods**

The heuristic shrinkage factor of Van Houwelingen and Le Cessie is defined as,

$$S_{VH}=1-\frac{p\text{ }}{\text{LR}}$$

*Equation (A1)*

where, $p$ is the total number of predictor parameters for the full set of candidate predictors (all those considered for inclusion in the model) and $\text{LR}$ is the likelihood ratio (chi-squared) statistic for the model. Riley et al. show that the heuristic shrinkage factor can be re-written as [16],

$$S_{VH}=1+\frac{p}{n \ln(1-R_{app}^{2})}$$

*Equation (A2)*

where $R_{app}^{2}$ is the apparent (‘app’) value of the Cox-Snell $R^{2}$ (a measure of proportion of variance exampled in the model development dataset) [21].

The log-likelihood of penalised regression approaches can be expressed generally in the form $\ln L_{model}-\lambda\mathrm{pen}\left( \beta\right)$, where $\mathrm{pen}\left( \beta\right)$ is the penalty term and λ is a non-negative tuning parameter, which controls the amount of shrinkage. The actual penalty term varies based on the penalised approach. For example, the penalised log-likelihood for the elastic net takes the form:

$${\ln L}_{p}={\ln L}_{model}-\lambda\left[ \left( 1-\alpha\right)\sum_{j=1}^{p} \beta_{j}^{2}+\alpha\sum_{j=1}^{p} \left| \beta_{j} \right| \right]$$

Here, ${\ln L}_{model}$ is the log-likelihood of the model (e.g. logistic regression) without penalisation, $\alpha$ is the mixing parameter and ranges between 0 and 1. An $\alpha=0$ is equivalent to ridge regression, and an $\alpha=1$ is equivalent to lasso. Whilst we could simultaneously tune over both $\alpha$ and λ, for simplicity in this paper we chose $\alpha=0.5$ for the elastic net applications.

**Further description of simulation study set-up**

After generating individuals’ values of twenty predictors ($x_{1}$ to $x_{20}$), the true outcome ($Y$ = 0 or 1) was generated for each individual based an underlying logistic regression model of

$$\ln\left( \frac{p}{1-p} \right)=\alpha+ LP$$

, where the linear predictor ($LP$) was

$LP=0.5x_{1}+0.3x_{2}+0.3x_{3}+0.25x_{4}+0.25x_{5}+\boldsymbol{0}(x_{6}+\cdots+x_{20}$)

and the intercept ($\alpha)$ was set to zero. The probability of an outcome event $p(Y=1|LP)$ was calculated for each individual using the linear predictor as $1/(1+\exp\left( -LP \right))$, and the true outcome (0 or 1) generated as a Bernoulli variable.

**Shrinkage is closer to 1 when R-squared is closer to 1**

Equation (A2) reveals that, for a particular number of participants ($n)$ and predictor parameters ($p)$, the heuristic shrinkage factor ($S_{VH})$ becomes closer to zero as the value of $R_{app}^{2}$ moves closer to zero. These analytical results are illustrated in the figure below, for a hypothetical prediction model of a continuous outcome developed using 5 predictor parameters; the estimate of $S_{VH}$ decreases exponentially toward 0 as the assumed $R_{app}^{2}$ moves from 1 to 0. For small reductions in $R_{app}^{2}$, the rate of change in the required $S_{VH}$ value is relatively steep for values of $S_{VH}$ < 0.8, and relatively flat when $S_{VH}$ > 0.9. This indicates that there is less uncertainty in the estimated value of $S_{VH}$ when the true $S$ is closer to 1, i.e. settings where overfitting is less of a concern.


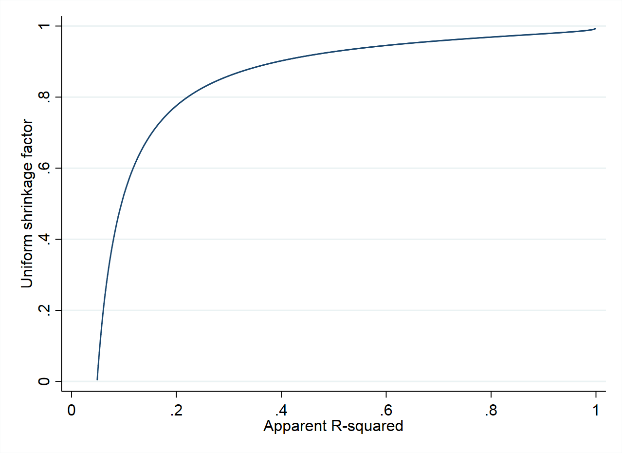


**Additional Figures and Tables**

Figure S1: Illustrating the variability in heuristic and bootstrap shrinkage factor by varying sample size

*
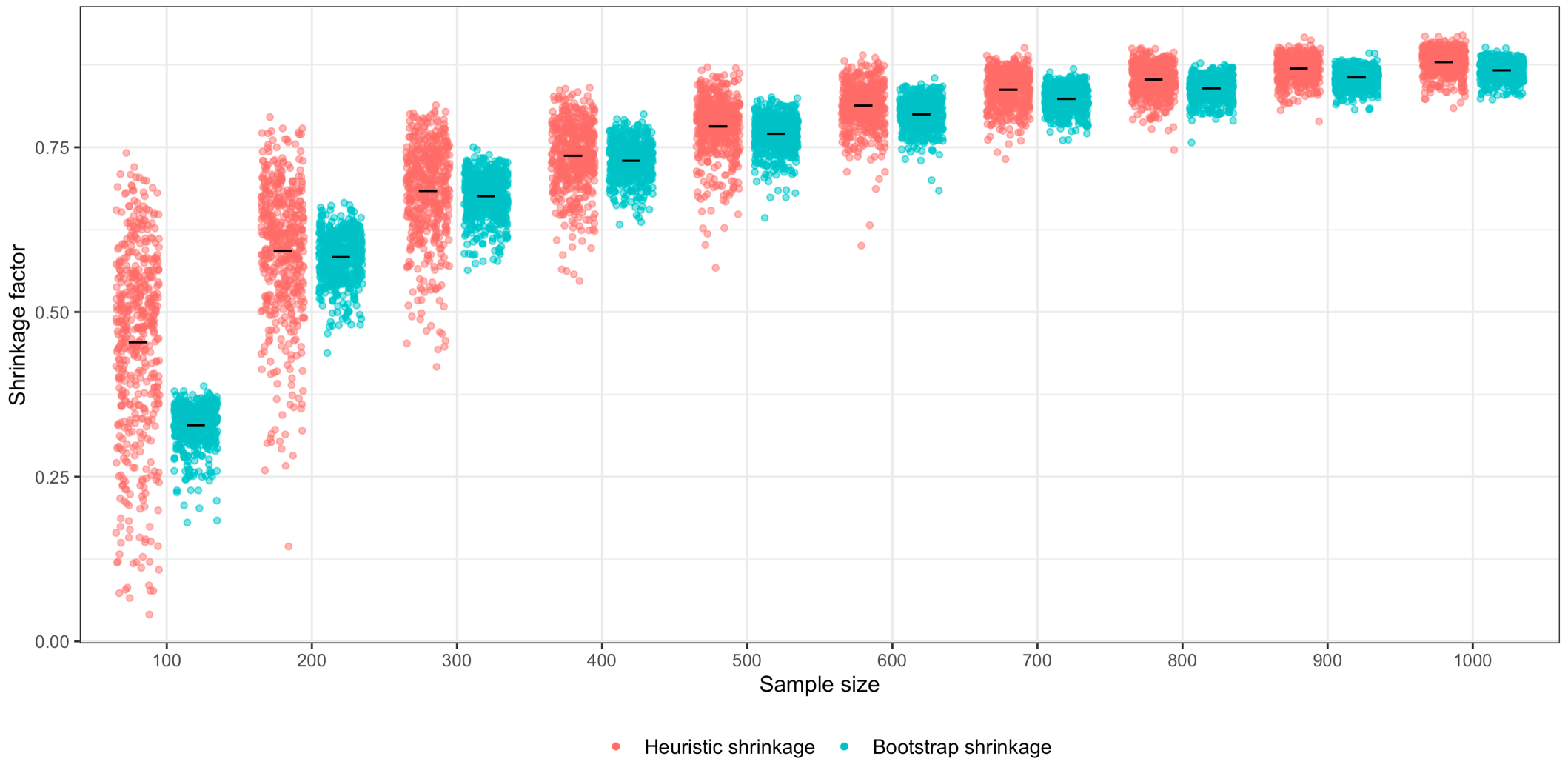
*

Figure S2: Apparent and validation c-index for models developed with no shrinkage (maximum likelihood) and penalised regression (ridge, elastic net, lasso)


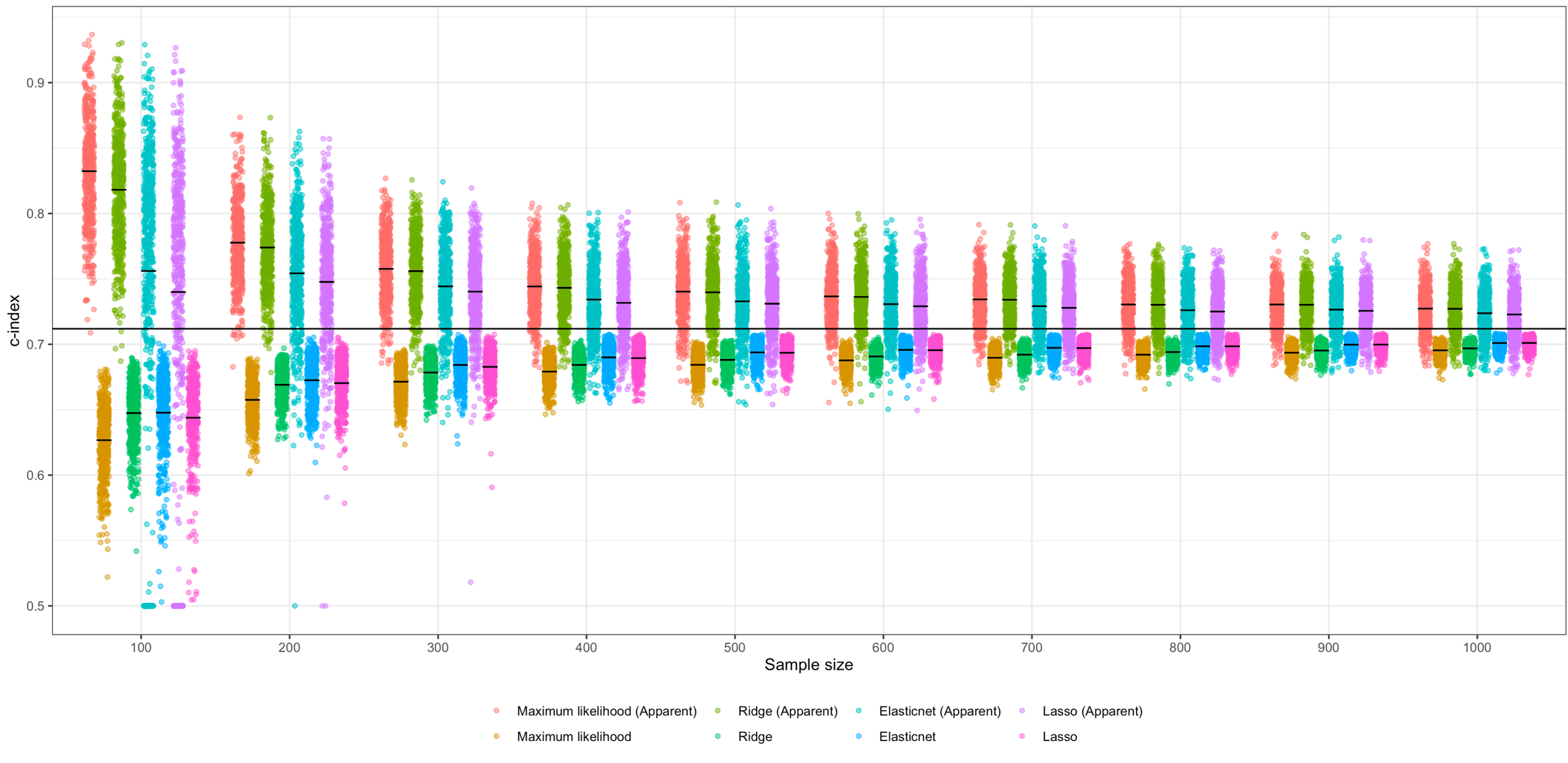


Figure S3: Calibration slope as estimated in the validation sample for models developed with no shrinkage (maximum likelihood), shrinkage (heuristic and bootstrap), and penalised regression (ridge, elastic net and lasso)


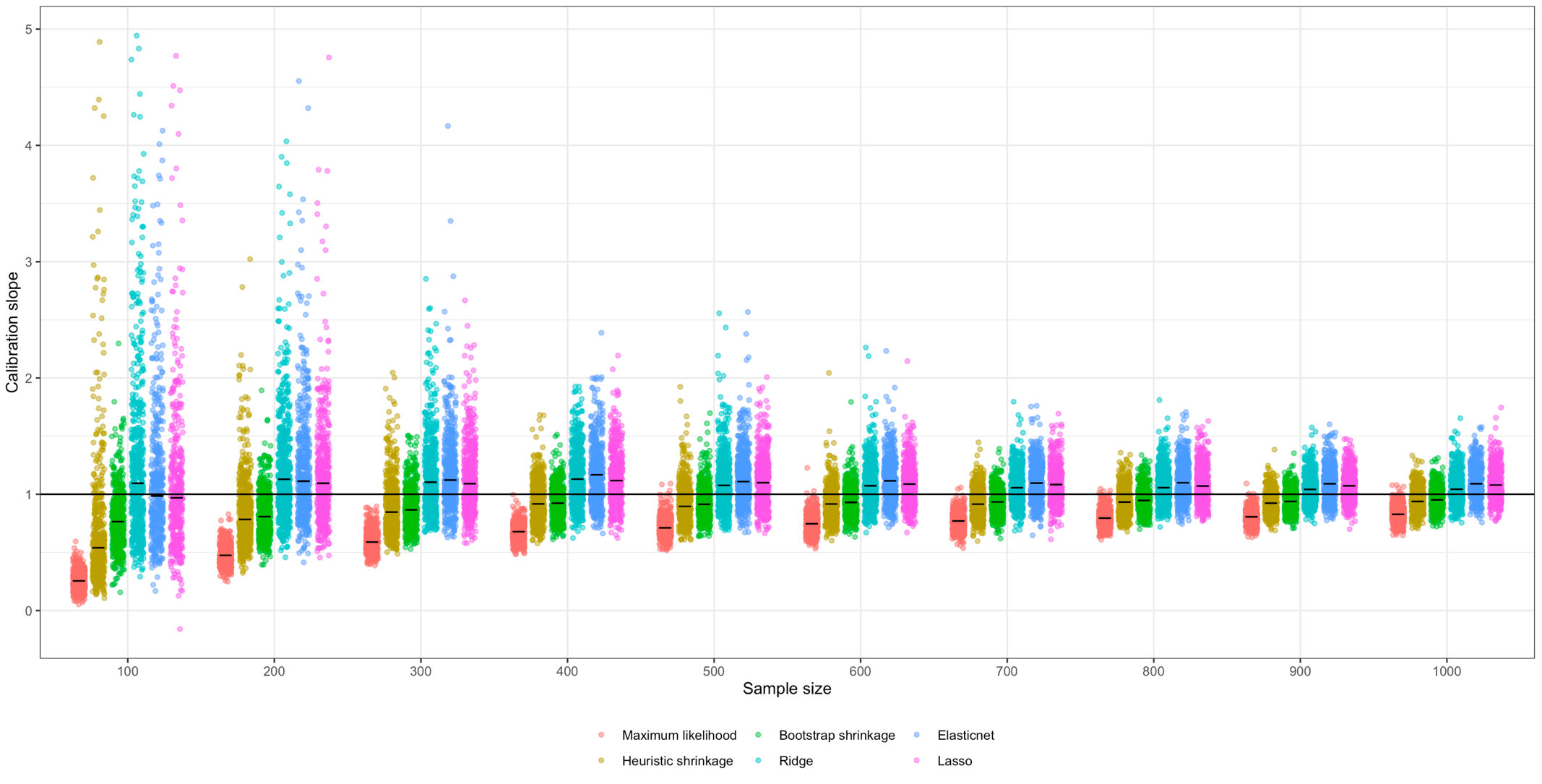


Figure S4: Standard deviation of the linear predictor for models developed with no shrinkage (maximum likelihood), and ridge regression (using 5-fold cross-validation, and bootstrap 5-fold cross-validation)


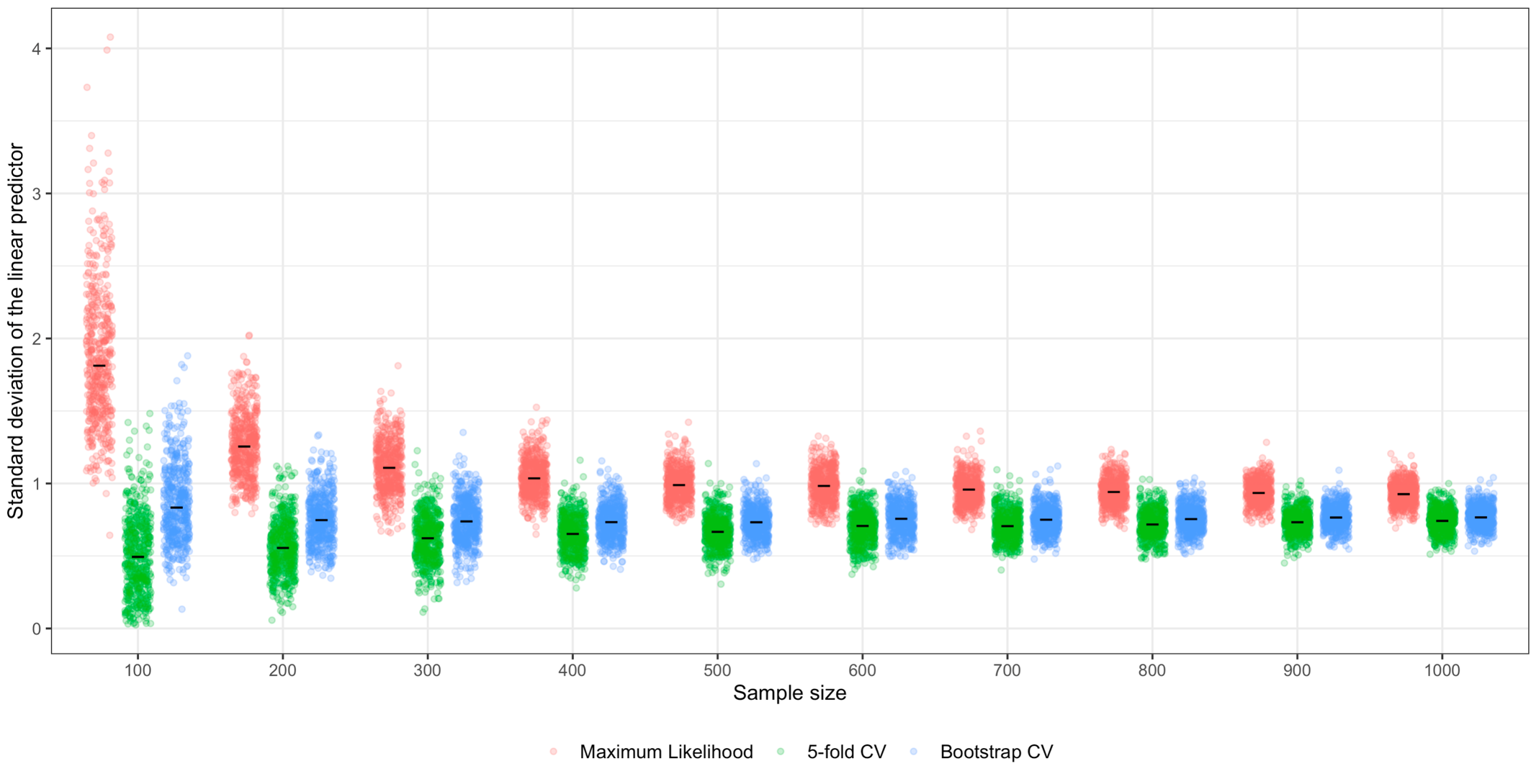


Figure S5: Box-plots and scatter plots showing variability in tuning parameter estimate $\lambda$ from model development using ridge regression (panel (a)), and the estimated c-index (panel (b)) and calibration slope (panel (c)) of the developed model when tested in the large validation data, across varying sample sizes for model development. For each sample size, 500 datasets were simulated as described in Section 2.3 and for each dataset a model was developed using ridge regression with either 5-fold cross-validation or bootstrap 5-fold cross-validation. In panels (b) and (c) comparison is also made to unpenalised maximum likelihood estimation. The horizontal lines in panels b and c are the large sample values. Note: Horizontal spread within each sample size grouping is just random jitter to aid display.

c


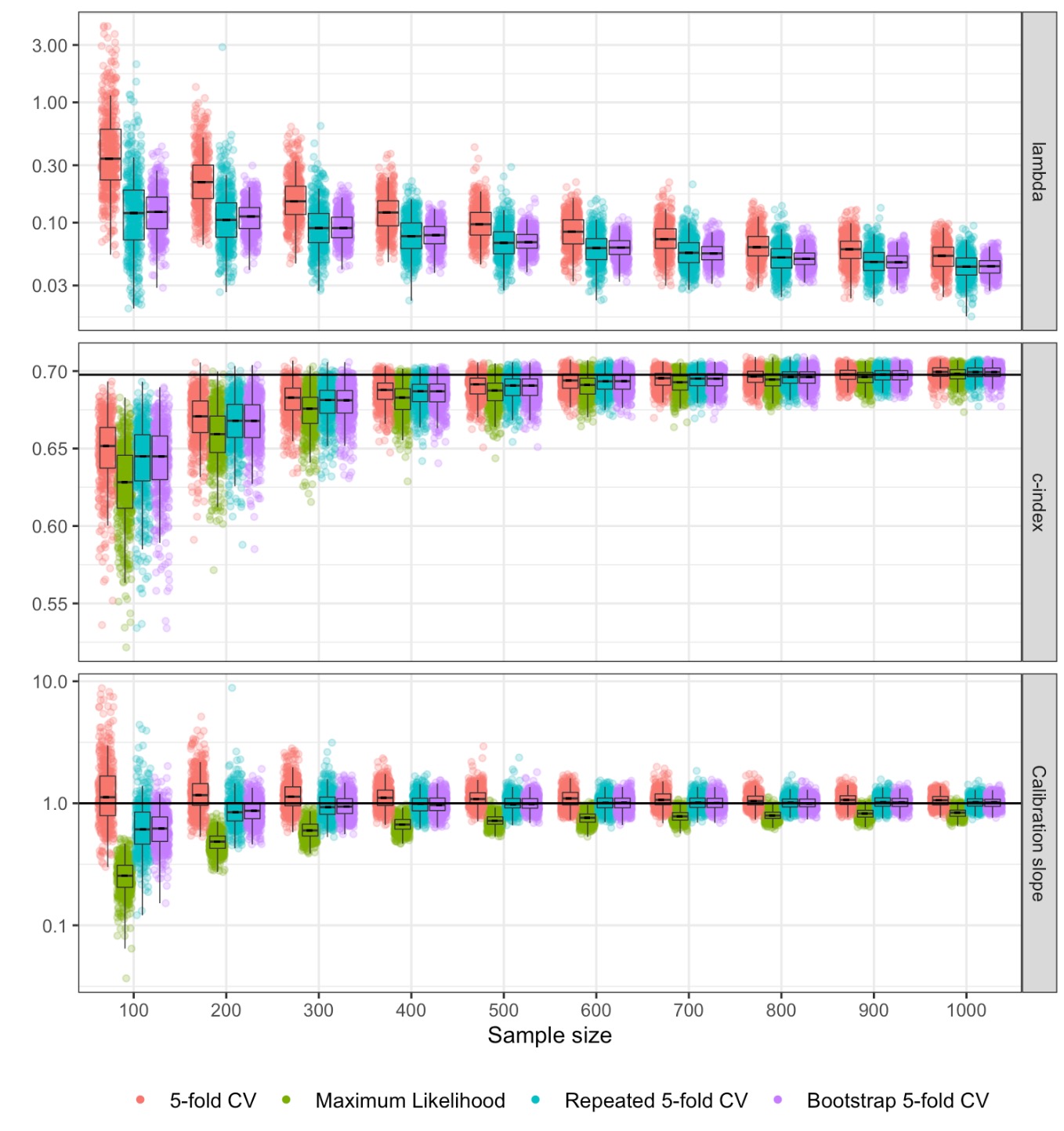


b

a

Table S1: Mean (sd) calibration slope and c-index in the validation data for models developed using ridge regression

| **n** | **Calibration Slope** | | | | | | | **c-index** | | | | | | |
| --- | --- | --- | --- | --- | --- | --- | --- | --- | --- | --- | --- | --- | --- | --- |
|  | **Maximum Likelihood** | **5-fold CV** | **Repeat 5-fold CV** | **Bootstrap 5-fold** | **10-fold CV** | **Repeat 10-fold CV** | **Bootstrap 10-fold CV** | **Maximum Likelihood** | **5-fold CV** | **Repeat 5-fold CV** | **Bootstrap 5-fold** | **10-fold CV** | **Repeat 10-fold CV** | **Bootstrap 10-fold CV** |
| 100 | 0.260  (0.081) | 1.324  (0.786) | 0.713  (0.447) | 0.655  (0.231) | 1.261  (0.752) | 0.672  (0.416) | 0.620  (0.219) | 0.627 (0.026) | 0.648  (0.023) | 0.641  (0.025) | 0.642  (0.025) | 0.648  (0.023) | 0.641  (0.025) | 0.641  (0.025) |
| 200 | 0.488  (0.088) | 1.286  (0.533) | 0.904  (0.286) | 0.886  (0.205) | 1.212  (0.454) | 0.870  (0.272) | 0.852  (0.194) | 0.658  (0.018) | 0.669  (0.016) | 0.666  (0.017) | 0.667  (0.016) | 0.669  (0.016) | 0.666  (0.017) | 0.666  (0.017) |
| 300 | 0.610  (0.098) | 1.215  (0.380) | 0.992  (0.276) | 0.971  (0.205) | 1.164  (0.348) | 0.959  (0.262) | 0.939  (0.195) | 0.674  (0.014) | 0.681  (0.012) | 0.679  (0.012) | 0.679  (0.012) | 0.681  (0.012) | 0.679  (0.012) | 0.679  (0.012) |
| 400 | 0.678  (0.092) | 1.152  (0.259) | 0.999  (0.186) | 0.992  (0.170) | 1.109  (0.233) | 0.972  (0.179) | 0.965  (0.163) | 0.681  (0.010) | 0.686  (0.009) | 0.685  (0.009) | 0.685  (0.009) | 0.686  (0.009) | 0.685  (0.009) | 0.685  (0.009) |
| 500 | 0.728  (0.082) | 1.128  (0.222) | 1.014  (0.170) | 1.011  (0.145) | 1.093  (0.196) | 0.989  (0.163) | 0.987  (0.140) | 0.686  (0.009) | 0.690  (0.008) | 0.689  (0.008) | 0.689  (0.008) | 0.690  (0.008) | 0.689  (0.008) | 0.689  (0.008) |
| 600 | 0.763  (0.086) | 1.108  (0.188) | 1.020  (0.153) | 1.018  (0.144) | 1.076  (0.172) | 0.996  (0.146) | 0.996  (0.139) | 0.690  (0.006) | 0.693  (0.006) | 0.693  (0.006) | 0.693  (0.006) | 0.693  (0.006) | 0.693  (0.008) | 0.693  (0.007) |
| 700 | 0.788  (0.083) | 1.096  (0.179) | 1.028  (0.146) | 1.022  (0.134) | 1.068  (0.162) | 1.007  (0.140) | 1.001  (0.130) | 0.692  (0.006) | 0.695  (0.006) | 0.694  (0.006) | 0.694  (0.006) | 0.694  (0.006) | 0.694  (0.006) | 0.694  (0.006) |
| 800 | 0.803  (0.076) | 1.071  (0.148) | 1.020  (0.128) | 1.014  (0.118) | 1.045  (0.136) | 1.000  (0.123) | 0.995  (0.114) | 0.692  (0.006) | 0.696  (0.006) | 0.696  (0.006) | 0.696  (0.006) | 0.696  (0.006) | 0.696  (0.006) | 0.695  (0.006) |
| 900 | 0.828  (0.079) | 1.079  (0.143) | 1.031  (0.125) | 1.027  (0.118) | 1.055  (0.135) | 1.013  (0.120) | 1.009  (0.115) | 0.696  (0.005) | 0.697  (0.005) | 0.697  (0.005) | 0.697  (0.005) | 0.697  (0.005) | 0.697  (0.005) | 0.697  (0.005) |
| 1000 | 0.838  (0.074) | 1.063  (0.124) | 1.023  (0.113) | 1.021  (0.107) | 1.042  (0.119) | 1.006  (0.109) | 1.004  (0.104) | 0.698  (0.005) | 0.699  (0.004) | 0.699  (0.004) | 0.699  (0.004) | 0.699  (0.004) | 0.699  (0.004) | 0.699  (0.004) |

Table S2: Median (Median Absolute Deviation) calibration slope and c-index in the validation data for models developed using ridge regression

| **n** | **Calibration Slope** | | | | | | | **c-index** | | | | | | | |
| --- | --- | --- | --- | --- | --- | --- | --- | --- | --- | --- | --- | --- | --- | --- | --- |
|  | **Maximum Likelihood** | **5-fold CV** | **Repeat 5-fold CV** | **Bootstrap 5-fold** | **10-fold CV** | **Repeat 10-fold CV** | **Bootstrap 10-fold CV** | **Maximum Likelihood** | **5-fold CV** | **Repeat 5-fold CV** | **Bootstrap 5-fold** | **10-fold CV** | **Repeat 10-fold CV** | **Bootstrap 10-fold CV** |  |
| 100 | 0.255  (0.078) | 1.104  (0.556) | 0.612  (0.264) | 0.622  (0.213) | 1.026  (0.484) | 0.577  (0.264) | 0.589  (0.197) | 0.631  (0.024) | 0.652  (0.019) | 0.645  (0.022) | 0.645  (0.021) | 0.652  (0.020) | 0.644  (0.023) | 0.644  (0.021) |  |
| 200 | 0.485  (0.082) | 1.164  (0.363) | 0.844  (0.207) | 0.868  (0.178) | 1.112  (0.323) | 0.822  (0.208) | 0.835  (0.171) | 0.661  (0.017) | 0.671  (0.015) | 0.668  (0.016) | 0.668  (0.016) | 0.670  (0.015) | 0.667  (0.016) | 0.667  (0.016) |  |
| 300 | 0.599  (0.096) | 1.133  (0.290) | 0.930  (0.212) | 0.939  (0.186) | 1.083  (0.263) | 0.900  (0.208) | 0.909  (0.182) | 0.674  (0.013) | 0.683  (0.010) | 0.681  (0.011) | 0.681  (0.011) | 0.683  (0.010) | 0.681  (0.011) | 0.681  (0.011) |  |
| 400 | 0.669  (0.091) | 1.109  (0.232) | 0.990  (0.174) | 0.970  (0.169) | 1.070  (0.212) | 0.958  (0.167) | 0.941  (0.159) | 0.681  (0.010) | 0.688  (0.008) | 0.687  (0.008) | 0.687  (0.008) | 0.688  (0.008) | 0.687  (0.008) | 0.687  (0.008) |  |
| 500 | 0.718  (0.074) | 1.084  (0.169) | 0.982  (0.132) | 0.987  (0.126) | 1.061  (0.156) | 0.958  (0.123) | 0.961  (0.124) | 0.687  (0.009) | 0.691  (0.007) | 0.691  (0.007) | 0.691  (0.007) | 0.691  (0.007) | 0.690  (0.007) | 0.690  (0.007) |  |
| 600 | 0.761  (0.087) | 1.096  (0.187) | 1.008  (0.135) | 1.010  (0.136) | 1.071  (0.168) | 0.984  (0.130) | 0.990  (0.131) | 0.690  (0.007) | 0.694  (0.006) | 0.693  (0.006) | 0.693  (0.006) | 0.694  (0.006) | 0.693  (0.006) | 0.693  (0.007) |  |
| 700 | 0.781  (0.081) | 1.066  (0.150) | 1.010  (0.133) | 1.004  (0.127) | 1.043  (0.143) | 0.991  (0.130) | 0.988  (0.124) | 0.693  (0.006) | 0.695  (0.006) | 0.695  (0.006) | 0.695  (0.006) | 0.695  (0.006) | 0.695  (0.006) | 0.695  (0.006) |  |
| 800 | 0.793  (0.068) | 1.045  (0.124) | 1.010  (0.112) | 0.998  (0.104) | 1.026  (0.117) | 0.991  (0.109) | 0.979  (0.099) | 0.695  (0.006) | 0.696  (0.005) | 0.696  (0.005) | 0.696  (0.005) | 0.696  (0.005) | 0.696  (0.005) | 0.696  (0.005) |  |
| 900 | 0.825  (0.078) | 1.065  (0.130) | 1.019  (0.118) | 1.018  (0.117) | 1.043  (0.125) | 1.001  (0.115) | 1.000  (0.114) | 0.697  (0.005) | 0.698  (0.004) | 0.698  (0.004) | 0.698  (0.004) | 0.698  (0.004) | 0.698  (0.004) | 0.698  (0.004) |  |
| 1000 | 0.838  (0.072) | 1.059  (0.121) | 1.019  (0.103) | 1.019  (0.101) | 1.034  (0.118) | 1.001  (0.100) | 1.001  (0.096) | 0.698  (0.005) | 0.699  (0.004) | 0.699  (0.004) | 0.699  (0.004) | 0.699  (0.004) | 0.699  (0.004) | 0.699  (0.004) |  |

Table S3: Median Absolute Deviation in Lambda for models developed using ridge regression

| **N** | **5-fold cross-validation** | | | **10-fold cross-validation** | | |
| --- | --- | --- | --- | --- | --- | --- |
|  | **Single CV** | **50-Repeat CV** | **Bootstrap CV** | **Single CV** | **50-repeated CV** | **Bootstrap** |
| 100 | 0.2299 | 0.0820 | 0.0541 | 0.1951 | 0.0706 | 0.0488 |
| 200 | 0.1053 | 0.0501 | 0.0336 | 0.0913 | 0.0454 | 0.0300 |
| 300 | 0.0597 | 0.0371 | 0.0244 | 0.0514 | 0.0339 | 0.0228 |
| 400 | 0.0420 | 0.0279 | 0.0191 | 0.0341 | 0.0252 | 0.0170 |
| 500 | 0.0305 | 0.0215 | 0.0129 | 0.0241 | 0.0190 | 0.0118 |
| 600 | 0.0281 | 0.0182 | 0.0121 | 0.0206 | 0.0166 | 0.0107 |
| 700 | 0.0197 | 0.0157 | 0.0109 | 0.0159 | 0.0147 | 0.0097 |
| 800 | 0.0167 | 0.0138 | 0.0082 | 0.0123 | 0.0114 | 0.0073 |
| 900 | 0.0146 | 0.0116 | 0.0080 | 0.0130 | 0.0103 | 0.0072 |
| 1000 | 0.0143 | 0.0104 | 0.0075 | 0.0116 | 0.0094 | 0.0056 |
