# Supplementary material for: Penalization and shrinkage methods produced unreliable clinical prediction models especially when sample size was small
Source: J Clin Epidemiol. 2021 Apr;132:88–96. doi: 10.1016/j.jclinepi.2020.12.005 (PMC8026952; doi:10.1016/j.jclinepi.2020.12.005)

**APPENDIX:**

**Further description on shrinkage and penalisation methods**

The heuristic shrinkage factor of Van Houwelingen and Le Cessie is defined as,

$$S_{VH}=1-\frac{p\text{ }}{\text{LR}}$$

*Equation (A1)*

where, $p$ is the total number of predictor parameters for the full set of candidate predictors (all those considered for inclusion in the model) and $\text{LR}$ is the likelihood ratio (chi-squared) statistic for the model. Riley et al. show that the heuristic shrinkage factor can be re-written as [16],

$$S_{VH}=1+\frac{p}{n \ln(1-R_{app}^{2})}$$

*Equation (A2)*

where $R_{app}^{2}$ is the apparent (‘app’) value of the Cox-Snell $R^{2}$ (a measure of proportion of variance exampled in the model development dataset) [21].

The log-likelihood of penalised regression approaches can be expressed generally in the form $\ln L_{model}-\lambda\mathrm{pen}\left( \beta\right)$, where $\mathrm{pen}\left( \beta\right)$ is the penalty term and λ is a non-negative tuning parameter, which controls the amount of shrinkage. The actual penalty term varies based on the penalised approach. For example, the penalised log-likelihood for the elastic net takes the form:

$${\ln L}_{p}={\ln L}_{model}-\lambda\left[ \left( 1-\alpha\right)\sum_{j=1}^{p} \beta_{j}^{2}+\alpha\sum_{j=1}^{p} \left| \beta_{j} \right| \right]$$

Here, ${\ln L}_{model}$ is the log-likelihood of the model (e.g. logistic regression) without penalisation, $\alpha$ is the mixing parameter and ranges between 0 and 1. An $\alpha=0$ is equivalent to ridge regression, and an $\alpha=1$ is equivalent to lasso. Whilst we could simultaneously tune over both $\alpha$ and λ, for simplicity in this paper we chose $\alpha=0.5$ for the elastic net applications.

**Further description of simulation study set-up**

After generating individuals’ values of twenty predictors ($x_{1}$ to $x_{20}$), the true outcome ($Y$ = 0 or 1) was generated for each individual based an underlying logistic regression model of

$$\ln\left( \frac{p}{1-p} \right)=\alpha+ LP$$

, where the linear predictor ($LP$) was

$LP=0.5x_{1}+0.3x_{2}+0.3x_{3}+0.25x_{4}+0.25x_{5}+\boldsymbol{0}(x_{6}+\cdots+x_{20}$)

and the intercept ($\alpha)$ was set to zero. The probability of an outcome event $p(Y=1|LP)$ was calculated for each individual using the linear predictor as $1/(1+\exp\left( -LP \right))$, and the true outcome (0 or 1) generated as a Bernoulli variable.

**Shrinkage is closer to 1 when R-squared is closer to 1**

Equation (A2) reveals that, for a particular number of participants ($n)$ and predictor parameters ($p)$, the heuristic shrinkage factor ($S_{VH})$ becomes closer to zero as the value of $R_{app}^{2}$ moves closer to zero. These analytical results are illustrated in the figure below, for a hypothetical prediction model of a continuous outcome developed using 5 predictor parameters; the estimate of $S_{VH}$ decreases exponentially toward 0 as the assumed $R_{app}^{2}$ moves from 1 to 0. For small reductions in $R_{app}^{2}$, the rate of change in the required $S_{VH}$ value is relatively steep for values of $S_{VH}$ < 0.8, and relatively flat when $S_{VH}$ > 0.9. This indicates that there is less uncertainty in the estimated value of $S_{VH}$ when the true $S$ is closer to 1, i.e. settings where overfitting is less of a concern.


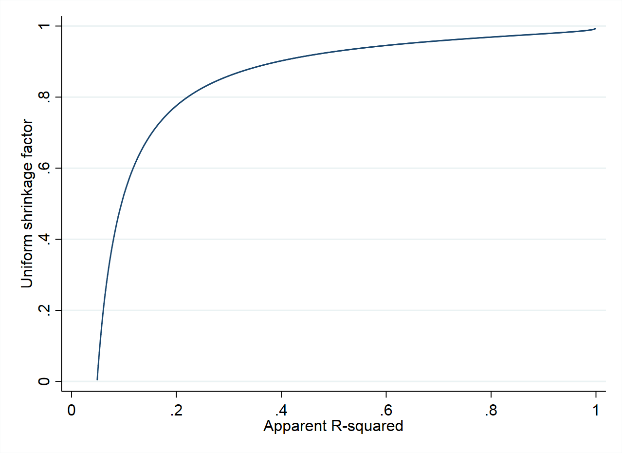

Supplement: Appendix [file mmc2.docx]
